# Progress in Medicine

**Published:** 1900-09-08

**Authors:** 


### Progress in Medicine.

### NEUROLOGY.

(*Continued from page* 369.)

**Optic Chiasma.**—Leszynsky[21] records three cases of lesion of the optic chiasma, and discusses the nature of the causes by which it may be produced. Four classes may be distinguished: (1) Associated with intracranial growths, and their concomitant symptomatology; (2) from enlargement of the pituitary body, as occurring in acromegaly; (3) in syphilitic basal meningitis; (4) from a circumscribed pathological process, which gradually produces complete atrophy of both optic nerves, without any cerebral symptoms whatever. Of these four classes, the first—destruction of the chiasma by extension of some tumour in adjacent structures—is by far the commonest; and syphilis is answerable for 20 per cent. The fourth class is very rare; in it the disease seems to be limited to the chiasma, the condition being ascribed to a local meningitis or periostitis, or to a non-progressive exostosis; but as yet no pathological investigation has taken place.

**Medullated Nerves.**—Wynne[22] has reinvestigated the structure of the supporting framework, in the meshes of which the fatty myelin of the sheath is contained. He finds that it consists of, first, two thin layers, one beneath the primitive sheath, the other around the axis cylinder; and, secondly, a chain of cones connected with both layers of protoplasm, the base of each cone being in the peripheral layer, and the apex in the central layer, and being composed of usually six segments. The neurokeratin network usually described Wynne believes to be artificially produced from the protoplasmic layer beneath the primitive sheath.

Mönckeberg and Bethe[23] have recently reinvestigated the earliest degenerative changes which occur in medullated nerve fibres. The normal axis cylinders consist of individual fibrils of sensibly uniform diameter lying in a homogeneous perifibrillar substance. The earliest pathological signs in degeneration consist of a swelling of the fibrils, which shortly afterwards break up into granular bodies of large size; later on these break up into smaller particles, which are eventually absorbed.

**Plantar Reflex.**—Walton and Paul[24] have recently recorded the results of examination of 700 cases to test the value of the phenomenon which was first described by Babinski, viz., that in pyramidal tract disease stroking the sole of the foot often produces extension of the great toe, with or without extension and separation of the other toes. These observers find that in health any one of the following conditions may appear on stroking the sole of the foot: Flexion of all toes, flexion of some (generally outer) toes, entire absence of movement on both sides (about 10 per cent.), flexion of all or some toes on one side and absence on the other. In early infancy no constant or characteristic movements of the toes appear, though extension is rather more frequent than flexion. The reflex described by Babinski —*i.e.*, deliberate extension of the great toe—appears in about 70 per cent. of hemiplegics and diplegics, and in approximately the same percentage of cases with disease involving the pyramidal tract in the spinal cord. This extensor reflex is never present in health, and its existence is very doubtful in either functional or organic nervous or other disease not implicating the pyramidal tract. This reflex is often the earliest to appear in pyramidal tract disease, *e.g.*, at the onset of a hemiplegic attack before the establishment of the exaggerated knee-jerk and ankle clonus; it may persist during a period when other reflexes are wanting, *e.g.*, when the knee-jerk and ankle-clonus are wanting on account of ankylosis, contracture, and muscular wasting, as in long-standing diplegia or of degenerative sequences, and in combined system disease. This reflex furnishes, therefore, a most important practical aid in diagnosis. The reflex very exceptionally appears in cases not conforming to recognised types of pyramidal disease (meningitis, hydrocephalus, poisoning as by alcohol, or uræmia). These instances are too few materially to impair the diagnostic value of the phenomenon; in fact, the extensor reflex alone should here rather lead us to suspect pyramidal involvement, as by œdema or indirect pressure.

**Neuromata.**—Bland Sutton[25] has recorded and figured some most interesting cases of neuromata and allied conditions. He shows that six apparently diverse conditions—neuromata, ganglionic neuromata, plexiform neuromata, molluscum fibrosum, in its solitary as well as its pendulous and nodular forms, sarcoma of nerve, and glioma of the central nervous system—are all due to a common cause, overgrowth of the delicate connective tissue which immediately surrounds the true nerve elements, the manifestation depending on the particular part of the nervous system affected, whether brain, spinal cord, large nerve trunks, terminal nerves in the skin, or sustentacular framework of the retina.

[21] Leszynsky, Jour. of Ment. and Nerv. Dis., Mar., 1900. [22] Wynne, Jour. of Anat. and Phys., Ap., 1900. [23] Mönckeberg and Bethe, Archiv. f. microscop. Anat., Vol. 54. [24] Walton and Paul, Jour. of Nerv. and Mental Dis., June, 1900. [25] Bland Sutton, Clin. Jour., June 13, 1900.

